# Multiple morphogenic culture systems cause loss of resistance to cassava mosaic disease

**DOI:** 10.1186/s12870-018-1354-x

**Published:** 2018-06-25

**Authors:** Raj Deepika Chauhan, Getu Beyene, Nigel J. Taylor

**Affiliations:** Donald Danforth Plant Science Center, St. Louis, MO USA

**Keywords:** Cassava, Cassava mosaic disease, Meristem tip culture, Organogenesis, Somatic embryogenesis

## Abstract

**Background:**

Morphogenic culture systems are central to crop improvement programs that utilize transgenic and genome editing technologies. We previously reported that CMD2-type cassava (*Manihot esculenta*) cultivars lose resistance to cassava mosaic disease (CMD) when passed through somatic embryogenesis. As a result, these plants cannot be developed as products for deployment where CMD is endemic such as sub-Saharan Africa or the Indian sub-continent.

**Result:**

In order to increase understanding of this phenomenon, 21 African cassava cultivars were screened for resistance to CMD after regeneration through somatic embryogenesis. Fifteen cultivars were shown to retain resistance to CMD through somatic embryogenesis, confirming that the existing transformation and gene editing systems can be employed in these genetic backgrounds without compromising resistance to geminivirus infection. CMD2-type cultivars were also subjected to plant regeneration via caulogenesis and meristem tip culture, resulting in 25–36% and 5–10% of regenerated plant lines losing resistance to CMD respectively.

**Conclusions:**

This study provides clear evidence that multiple morphogenic systems can result in loss of resistance to CMD, and that somatic embryogenesis *per se* is not the underlying cause of this phenomenon. The information described here is critical for interpreting genomic, transcriptomic and epigenomic datasets aimed at understanding CMD resistance mechanisms in cassava.

**Electronic supplementary material:**

The online version of this article (10.1186/s12870-018-1354-x) contains supplementary material, which is available to authorized users.

## Background

Cassava mosaic disease (CMD) is endemic throughout Sub-Saharan Africa and the Indian sub-continent. Effective resistance to the whitefly-vectored geminiviruses that cause CMD is essential to secure yields for cassava farmers across these regions. Three genetic sources of CMD resistance, i.e. CMD1, CMD2 and CMD3, have been identified. CMD1 resistance was introgressed from *Manihot glaziovii* and understood to be multigenic and recessive, while CMD2 is monolocus, dominant in nature and was identified in landraces collected in Nigeria and Benin/Togo [1, 2]. CMD3 carries the CMD2 locus plus an additional QTL [3]. In all three resistance types, the underlying genes and molecular mechanisms remain unknown. We recently reported that all plants of CMD2-type cultivars regenerated through somatic embryogenesis lose resistance to CMD and develop severe mosaic symptoms when inoculated with infectious geminivirus clones in the greenhouse, and when exposed to viliferous whiteflies in the field. Cultivars tested that carry CMD1 and CMD3 resistance mechanisms did not suffer from this phenomenon with plants regenerated through somatic embryogenesis remaining resistant to CMD [4].

Uniform and consistent loss of a major trait such as virus resistance in multiple cultivars by simple passage through embryogenesis is unique in the literature. Increasing understanding of why CMD2 resistance is compromised in this manner is imperative to the success of cassava enhancement programs. In general, phenotypic variations in plants recovered through tissue culture can be attributed to genetic or epigenetic changes. Changes in the DNA methylation status of the cassava genome were reported in plants regenerated via meristem tip culture by Kitimu et al. [5].

The single locus, dominant nature of CMD2 makes it highly favored by breeders as a source of resistance to generate improved planting materials [6]. Reliance on a single gene mechanism, however, risks evolution of the pathogen to overcome the resistance. Indeed, breakdown of CMD2-mediated resistance was reported recently under greenhouse conditions by Ndunguru, et al. [7]. Advanced biotechnologies in cassava rely on induction of somatic embryogenesis to generate the totipotent tissues utilized for transgene integration and delivery of gene editing reagents [8, 9]. Genetic modification in this manner must be achieved without losing resistance to CMD, a trait that is essential in all enhanced cassava germplasm intended for deployment in Africa and India.

We report here further evidence for loss of functional CMD2-mediated resistance when tissues are passed through morphogenic culture systems. In addition to somatic embryogenesis, information is presented describing the effects of caulogenesis and meristem tip culture on loss of resistance to CMD in regenerated plants.

## Methods

### Media composition and culture conditions

Compositions of culture media used in this study followed Chauhan, et al. [10] for induction of organized embryogenic structures (OES) and friable embryogenic callus (FEC); Chauhan and Taylor [11] for organogenesis; and the International Institute of Tropical Agriculture Handbook [12] for meristem tip culture. Media components, antibiotics, growth regulators and additives were procured from Sigma (St. Louis, MO, USA). Meta-topolin (*m*T) used for regeneration of plants through organogenesis was obtained from Duchefa Biochemie, The Netherlands. All in vitro cultures were incubated at 28 ± 1^o^ C with 16 h light/ 8 h dark photoperiod under fluorescent lamps at 75 μmol m^− 2^ s^-^^1^ unless otherwise specified.

### Plant material and gene constructs

In vitro shoot cultures of CMD1-type cassava cultivar TMS 30572, CMD2-type cultivars TME 419, TME B7, CMD3-type cultivars TMS 98/0581, TMS 98/0505, TMS 96/1632 and other cultivars with unknown CMD-types NR 03/0155, TMS 98/0002, TMS 01/0040, TMS 92/0057, TMS 01/1206, TMS 91/02324, TMS 98/2132, TMS 92/0326, TMS 01/1371, TMS 95/0289 and 60444 were obtained from IITA, Nigeria (Table 1). Stem cuttings of CMD1-type cultivars NASE 3, NASE 14 and CMD2-type cultivars TME 14, TME 204 were imported from the National Crops Resources Research Institute (NaCRRI), Uganda, and TME 7 from IITA collected from farmer fields in Nigeria*.* Stem cuttings were established under in vitro conditions at the Donald Danforth Plant Science Center (DDPSC), St. Louis, MO, USA. Axillary buds that developed from the stems were excised and established in tissue culture following methods described by Taylor, et al. [13] and Chauhan, et al. [10]. CMD susceptible plants of TME 204 were obtained by regeneration from friable embryogenic callus (FEC-TME 204) [10]; [4] and served as known negative controls for greenhouse trials.

**Table 1:** Induction of organized embryogenic structures (OES), friable embryogenic structures (FEC) from cassava cultivars and response to MeSPY1-VIGS cassava mosaic disease challenge

Cultivar name	Resistance type	Organized embryogenic structures (OES) induction frequency (%)	Friable Embryo-genic Callus (FEC) induction (Yes/No)	Number of dead plants/total plants challenged with MeSPY1-VIGS		Resistance/ susceptibility to cassava mosaic disease	
				Wildtype	OES-derived	Wildtype	OES-derived
NASE 3	CMD1	24	No	1/8	3/8	Resistant	Resistant
NASE 14	CMD1	81	Yes	0/9	0/10	Resistant	Resistant
TMS 30572	CMD1	28	No	2/9	1/8	Resistant	Resistant
TME 204	CMD2	81	Yes	1/9	7/7^a^	Resistant	Susceptible
TME B7	CMD2	95	Yes	1/9	6/6	Resistant	Susceptible
TME 419	CMD2	58	Yes	7/9	6/6	Susceptible	Susceptible
TMS 96/1632	CMD3	63	No	0/6	0/9	Resistant	Resistant
TMS 98/0505	CMD3	55	Yes	0/8	0/9	Resistant	Resistant
TMS 98/0581	CMD3	66	No	0/7	0/10	Resistant	Resistant
TMS 92/0326	Unknown	89	Yes	1/8	1/7	Resistant	Resistant
TMS 92/0057	Unknown	76	No	0/12	0/5	Resistant	Resistant
TMS 95/0289	Unknown	3	No	3/11	NA	Resistant	Not tested
TMS 98/2132	Unknown	79	No	0/6	0/4	Resistant	Resistant
NR03/0155	Unknown	53	No	0/9	0/8	Resistant	Resistant
TMS 91/02324	Unknown	53	Yes	0/10	0/9	Resistant	Resistant
TMS 98/0002	Unknown	78	No	0/10	0/10	Resistant	Resistant
TMS 01/0040	Unknown	59	Yes	0/10	0/9	Resistant	Resistant
TMS 01/1206	Unknown	66	Yes	0/7	0/8	Resistant	Resistant
TMS 01/1371	Unknown	64	No	0/8	0/9	Resistant	Resistant
Mbundamali	Unknown	Not determined	Not tested	10/10	5/5	Susceptible	Susceptible
60444	Susceptible	90	Yes	9/9	NA	Susceptible	Not tested

^a^FEC derived TME 204 used as control (FEC-TME 204)

*Agrobacterium tumefaciens* strain LBA4404 harboring a pCAMBIA2300-based binary vector containing the enhanced green fluorescent protein gene (*egfp)* under control of the *Cauliflower mosaic virus* (CaMV) 35S promoter was used for transformation experiments, following procedures described by Chauhan, et al. [10].

### Production of organized embryogenic structures (OES) and plant regeneration

Induction of organized embryogenic structures (OES) was performed as described by Taylor, et al. [13] and Chauhan, et al. [10]. Immature leaf lobe explants were excised from 4- to 6-week-old micropropagated shoot cultures and placed on DKW/Juglans basal salts [14] (*Phyto*Technology Laboratories, Kansas, USA) plus Murashige and Skoog (MS) [15] vitamins, supplemented with 2% *w*/*v* sucrose and 50 μM picloram (DKW 50P). Cultures were incubated in the dark at 28 °C for 4 weeks. Eight leaf lobe explants were cultured per plate with five plates per cultivar, and experiments replicated three times. The number of explants forming OES was assessed 5 weeks after explanting.

Plants were regenerated 8–10 weeks after leaf lobe explant initiation by excising OES from the non-embryogenic tissues and subculture onto MS media containing 2% sucrose *w*/*v* (MS2) and 2 μM *m*T solidified with 0.22% *w/v* gelzan [11]. Between eight and 10 colonies of OES were cultured in each plate. After 4 weeks, individual cotyledon stage embryos were separated from each other and subcultured onto fresh media of the same type. Germinating shoots possessing two to three true leaves were transferred for rooting to MS media supplemented with 2% *w*/*v* sucrose and solidified with 0.8% *w/v* Noble agar.

### *Agrobacterium*-mediated transformation and plant regeneration

Friable embryogenic callus (FEC) produced from six cultivars TMS 98/0505, TMS 01/0040, TMS 01/1206, TMS 91/02324, TME B7 and TME 419 was transformed with *Agrobacterium tumefaciens* strain LBA4404 harboring a pCAMBIA2300-based binary vector carrying *egfp* following the method described by Chauhan, et al. [10]. *Agrobacterium* suspension at an OD_600_ of 0.05 was used to inoculate FEC and the cultures were kept at 22 °C under constant light. Three to 4 days after the inoculation, the *Agrobacterium* was washed off from the FEC. The tissues were then selected on media containing 27.5 μM paromomycin followed by transfer to embryo maturation media containing 45 μM paromomycin. The cotyledon stage embryos were germinated and rooted on selection free media. GFP-expressing tissues were visualized under a Nikon C15304 dissecting microscope equipped with an excitation filter of 460–500 nm and barrier filter, 510 LP at different stages after transformation and scored as described by Chauhan, et al. [10]. Three replicates were established per cultivar for each treatment and transformation experiments repeated two times. Non-transgenic plants for use as negative controls were recovered from non-transformed FEC.

### Regeneration of cassava plants through organogenesis

Plants of CMD2-type cultivars TME 7 and TME 204 were regenerated from leaf-petiole explants following Chauhan and Taylor [11]. Leaf-petiole explants were excised from mother plants pre-treated with 2 μM *m*T for 4 weeks, cultured on MS medium supplemented with 2% *w*/*v* sucrose, 1 μM 2,4-D and 1 μM *m*T for 7 days, followed by transfer to MS2 medium containing 6 μM *m*T. Tissues were subcultured onto fresh media of the same type every 2–3 weeks. Regenerated shoots 2.0 to 2.5 cm in length were transferred to MS2 media for rooting and plantlet establishment.

### Regeneration of cassava plants through meristem tip culture

Plants of CMD2-type cultivars TME 7, TME 14, TME 204, CMD1-type cultivar TMS 30752 and CMD3-type cultivar TMS 98/0505 were regenerated through meristem tip culture following the method described by IITA [12]. Six- to eight-week-old in vitro micropropagated mother plants cultured on MS media supplemented with 2% *w*/*v* sucrose (MS2) and solidified with 0.8% *w/v* noble agar were used as the explant source. Leaf primordia were removed from the shoot tip using a hypodermic needle under a stereomicroscope (Olympus SMZ51) until the meristematic dome was visible. The meristem tip (~ 0.5 mm in size) was excised and placed on MS basal media supplemented with 0.1 g/l inositol, 0.08 g/l adenine sulfate, 1.07 μM NAA (1-napthalene acetic acid), 0.22 μM BAP (6-benzylaminopurine), 0.23 μM GA_3_(gibberellic acid), 3% *w*/*v* sucrose and solidified with 0.4% *w/v* Noble agar. Cultures were incubated in the dark for two to 4 weeks at 28 ± 1^o^ C. Regenerating shoots were rooted on MS2 media. Between 18 and 40 meristem tip explants were excised and cultured for each cultivar with the number of explants inducing shoots suitable for transferring to rooting media assessed after 5 weeks in culture.

### Inoculation of the plants with geminiviruses in the greenhouse

Plants that recovered through all morphogenic pathways were propagated along with the controls on MS2 media and solidified with 0.22% *w*/*v* gelzan. After three to 4 weeks of culture, plantlets were transferred to Fafard 51 growing mixture in 3-inch pots and placed on a mist bench at 100% relative humidity for 7 days followed by transfer to the open bench at 28 ± 1 °C day/ 25 ± 1° C night temperature in a 14 h light/10 h dark photoperiod at 380 to 420 μmolm^− 2^ s^-^^1^ irradiance and 80–90% relative humidity and allowed to grow for 3 weeks [13]. Plants 8 to 9 cm in height were transferred to a greenhouse and grown at a 32^o^ C day/ 27° C night cycle with 70–95% relative humidity.

A rapid VIGS-based screening method developed by Beyene et al. [16] was employed to determine the CMD status of the plants recovered from OES and meristem tip culture. Four- to six-week-old plants were inoculated with plasmid DNA of MeSPY1 (*Manihot esculenta* SPY) -VIGS and the DNA-B component of *East African cassava mosaic virus* (EACMV-K201) using a Helios® Gene Gun (BioRad, Hercules, California). This causes silencing of *MeSPY* which leads to shoot-tip necrosis and death of the plant in CMD-susceptible cassava plants within 2-4 weeks of inoculation whereas the CMD-resistant plants remain healthy. The shoot-tip necrosis and death of plants were scored commencing 14 days after inoculation.

Plants recovered from FEC, meristem tip culture and organogenesis were inoculated with cassava geminiviruses following Beyene, et al. [4]. Four-week-old greenhouse-grown plants were inoculated with infectious clones of *East African cassava mosaic virus* (EACMV-K201) DNA-A GenBank: AJ717541 and DNA-B GenBank: AJ704953) [17, 18] and *African cassava mosaic virus* Cameroon strain (ACMV-CM) DNA-A GenBank AF112352 and DNA-B GenBank AF112353 [19] using a Helios® Gene Gun (BioRad, Hercules, CA, USA). Inoculated plants were assessed for CMD symptoms starting 7 days post inoculation (DPI), with symptom severity scored on a scale of 0–5 [20] twice per week.

## Results

### Screening cassava cultivars for CMD resistance after passage through somatic embryogenesis

We previously reported that CMD2-type cassava plants that had been regenerated through somatic embryogenesis lose resistance to CMD but that no such effect is observed in cultivars carrying CMD1 and CMD3 resistance mechanisms [4]. To investigate this phenomenon further, 21 cassava cultivars (Table 1) from East and West Africa were passed through somatic embryogenesis by inducing OES from leaf explants [10, 13]. Plants regenerated from OES were challenged with an infectious VIGS clone of EACMV-K201 modified to carry sequences that target *MeSPY1*. Plants with functional resistance to geminviruses recover from this inoculation, while shoot-tip of susceptible plants wilt and die within two to 4 weeks after inoculation [16]. Plants were also inoculated with the infectious clone of EACMV- K201 [4]. Similar results were obtained from both CMD challenge methods.

**Fig. 1:**
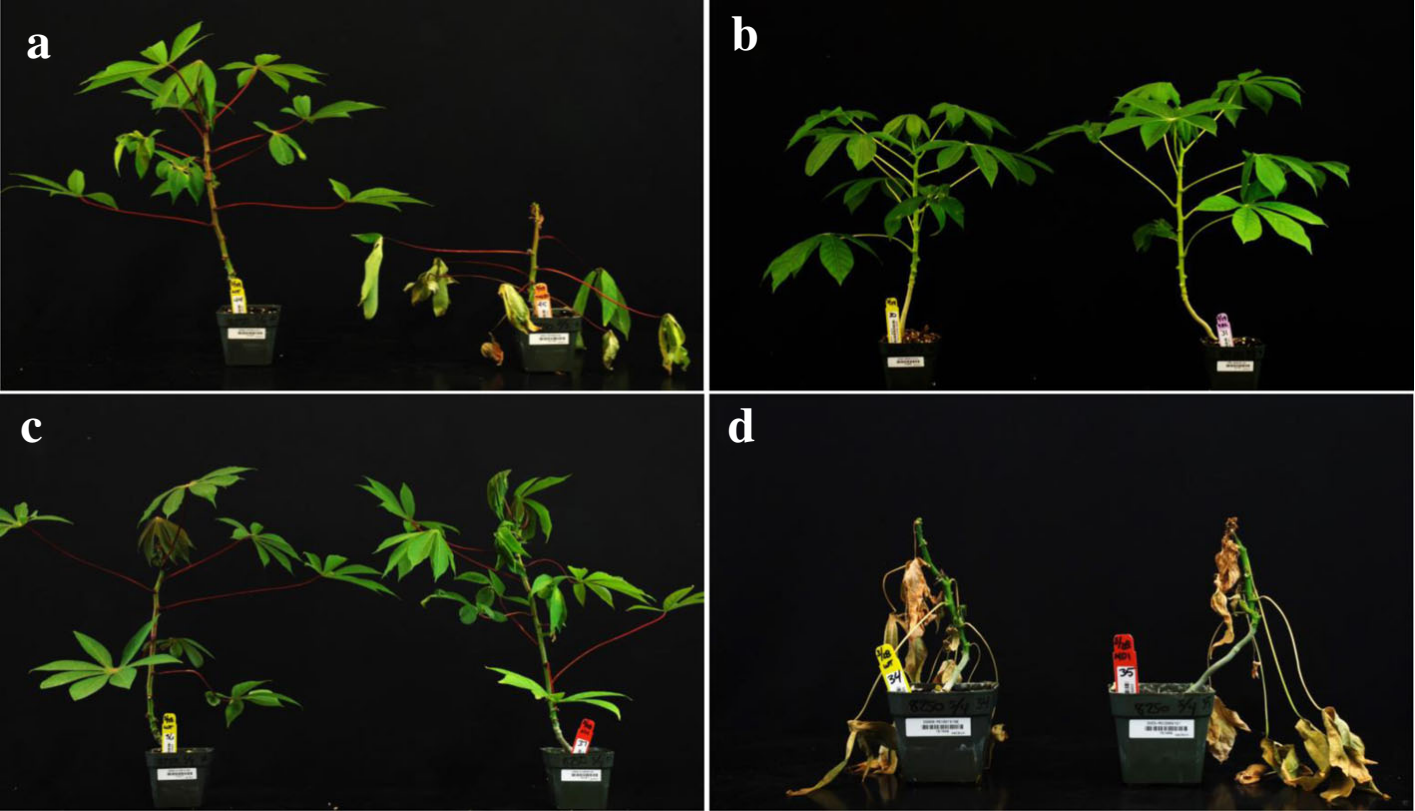
Response of wild-type (left) and organized embryogenic structures (right) derived plants to inoculation with MeSPY1-VIGS to determine resistance to cassava mosaic disease. Silencing of *MeSPY* using MeSPY1-VIGS leads to shoot-tip necrosis and death of CMD susceptible cassava plants within 2–4 weeks after inoculation. **a** TME B7. **b** TMS 98/0002. **c** NASE 14. **d** Mbundamali

All 21 cultivars tested underwent somatic embryogenesis to produce OES, with efficiencies varying from as high as 90% in 60444, to only 3% in TMS 95/0289 (Table 1). Plants were regenerated for all cultivars (except TMS 95/0289), established in the greenhouse and subjected to inoculation with MeSPY1-VIGS. Wild-type plants of the known CMD2-types TME 204 and TME 7 demonstrated resistance to CMD and survived the MeSPY1-VIGS challenge. Conversely, shoot-tips of plants of CMD2-type cultivars regenerated from OES started to wilt 12–14 DPI and subsequently died (Fig. 1). As consistently observed in our laboratory, wild-type plants of the CMD2-type cultivar TME 419 possess low-level resistance to infection with the infectious clone EACMV-K201, although it does possess robust resistance to ACMV (data not shown). Wild-type plants of cassava cultivars Mbundamali and 60444 are CMD susceptible and remained so after regeneration through somatic embryogenesis. The remaining 15 cultivars, whether carrying CMD1, CMD3 or unknown types of resistance to CMD, remained fully resistant to inoculation with MeSPY1-VIGS after passage through somatic embryogenesis (Table 1).

**Fig. 2:**
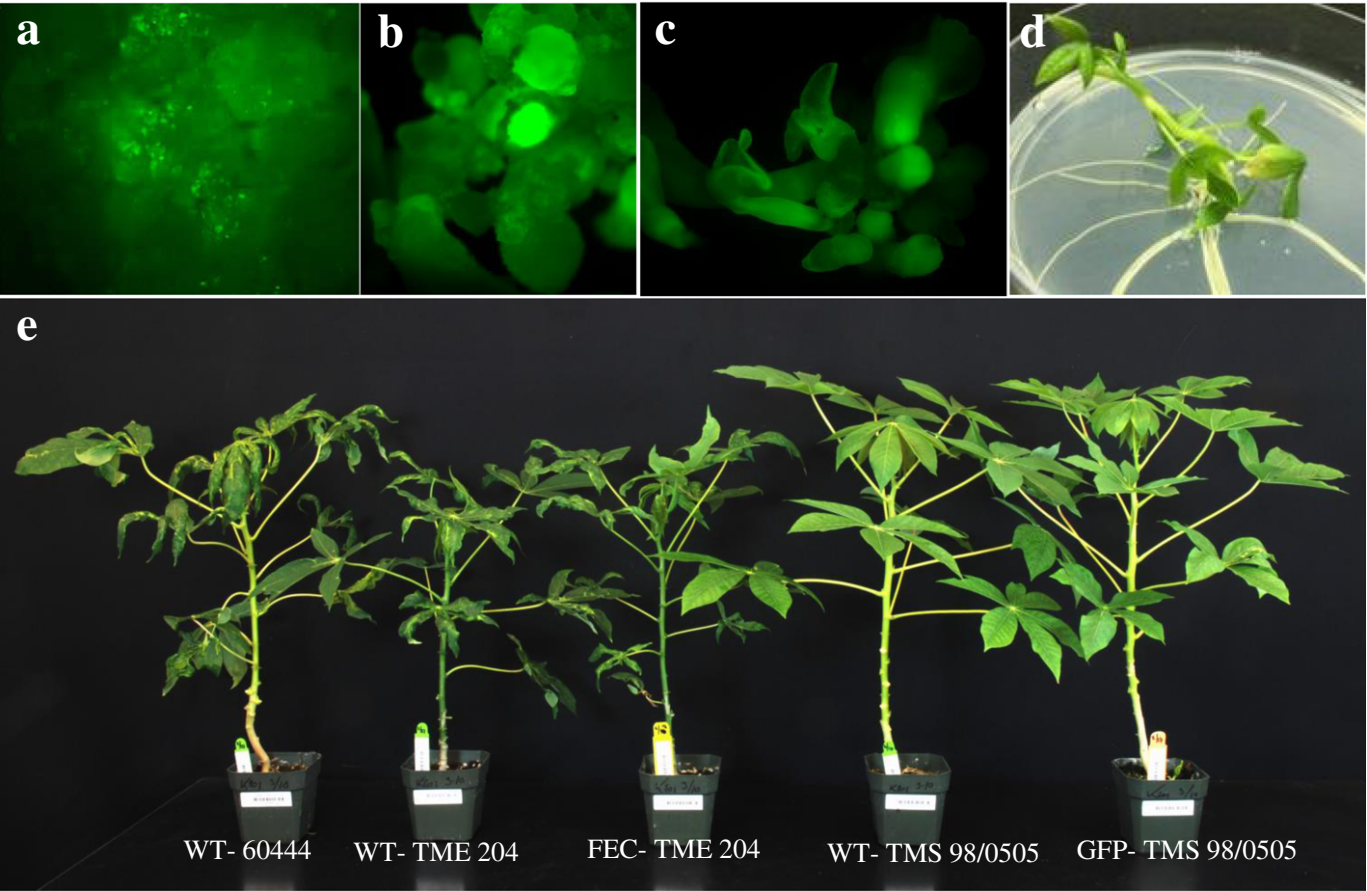
*Agrobacterium*-mediated genetic transformation of TMS 98/0505 and response of transgenic plants to inoculation with the infectious geminivirus clone EACMV-K201. **a** transient GFP expression after 4 days co-culture with *A. tumefaciens.* **b** GFP-expressing callus line. **c** GFP-expressing somatic embryos on regeneration media. **d** Transgenic rooted plant. **e** Response of transgenic and micropropagated wild-type plants to EACMV-K201 at 33 days post inoculation

**Fig. 3:**
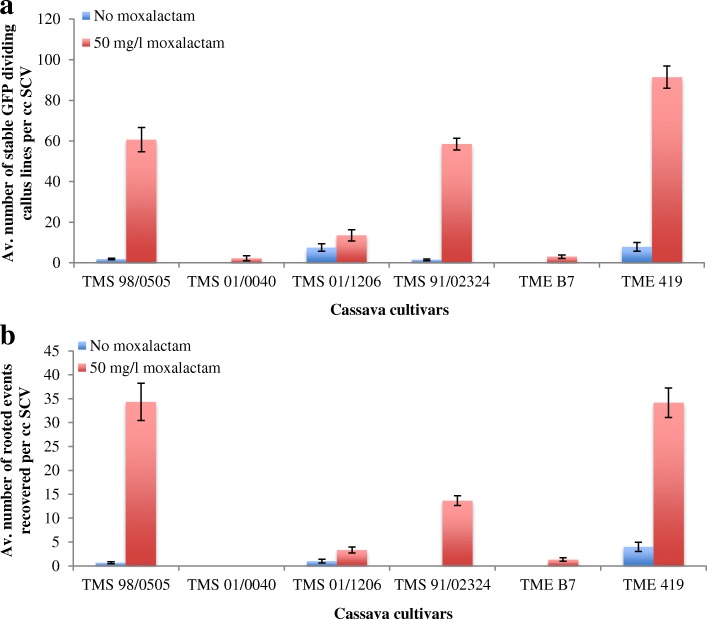
Stable GFP-expressing transgenic events recovered from friable embryogenic callus (FEC) of different cassava cultivars. **a** Average number of GFP positive callus lines obtained after 5 weeks of co-culture. **b** Average number of GFP positive rooted events obtained after 4–5 months of co-culture. Values are Average ± SE, Number of experiments done = 2 and Replications = 3 per experiment

**Fig. 4:**
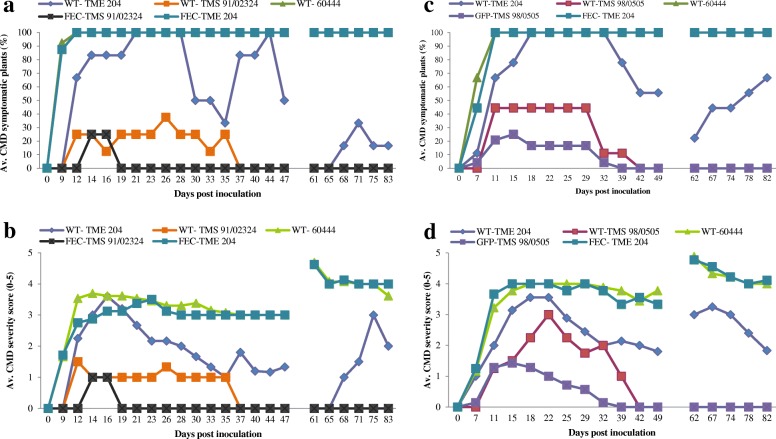
Response of non-transgenic and transgenic cassava plants to inoculation with the infectious geminivirus clone EACMV-K201. Non-transgenic and transgenic plants of TMS 91/02324 and CMD3-type cultivar TMS 98/0505, respectively, were generated from FEC. **a** Percentage of cassava mosaic disease (CMD) symptomatic plants of FEC-derived and micropropagated TMS 91/02324. **b** Average CMD symptom severity scores (scale 0–5) on FEC-derived and micropropagated TMS 91/02324. **c** Percentage of CMD symptomatic plants of transgenic GFP expressing TMS 98/0505 and wild-type TMS 98/0505. **d** Average CMD symptom severity scores (scale 0–5) on GFP-expressing TMS 98/0505 and wild-type TMS 98/0505. Plant stems were cut back 48 days after biolistic inoculation and CMD assessed on new leaf growth. Breaks in the *x* axis indicate a lapse in shoot regrowth after stem cut-back

FEC is the preferred target tissue for genetic transformation and is being adapted for the application of gene editing in cassava [8–10]. OES from all 21 cultivars shown in Table 1 were subcultured onto Gresshoff and Doy [21] -based medium in order to produce FEC. FEC was successfully generated from 10 cultivars, including six West African varieties that have not been reported previously (Table 1). Transgenic plant production was attempted by *Agrobacterium*-mediated transformation of FEC in the six cultivars TMS 98/0505, TMS 01/0040, TMS 01/1206, TMS 91/02324, TME B7 and TME 419. GFP-expressing callus lines were recovered in all cases (Figs. 2 and 3). As described previously [10], transformation was significantly more efficient if moxalactam was included in the culture medium prior to co-culture with *Agrobacterium* (Fig. 3). Transgenic plants were recovered from cultivars TMS 98/0505, TMS 01/1206 and TMS 91/02324, in addition to TME 419 and TME B7. FEC-derived plants of TMS 91/02324 (Fig. 4a & b) and TMS 98/0505 and transgenic GFP-expressing plant lines of TMS 98/0505 (Fig. 4c & d) were established in the greenhouse and inoculated with EACMV-K201 (Fig. 2). Of four TMS 91/02324 FEC-derived, five TMS 98/0505 FEC-derived, and 24 transgenic GFP-expressing TMS 98/0505 independent lines challenged, all plants recovered to display no mosaic symptoms within five to 6 weeks after challenge (Fig. 4). This data indicates that resistance to CMD was retained through all stages of somatic embryogenesis (OES and FEC), genetic transformation and plant regeneration (Fig. 2e).

### Effect of organogenesis and meristem tip culture on CMD resistance

**Fig. 5:**
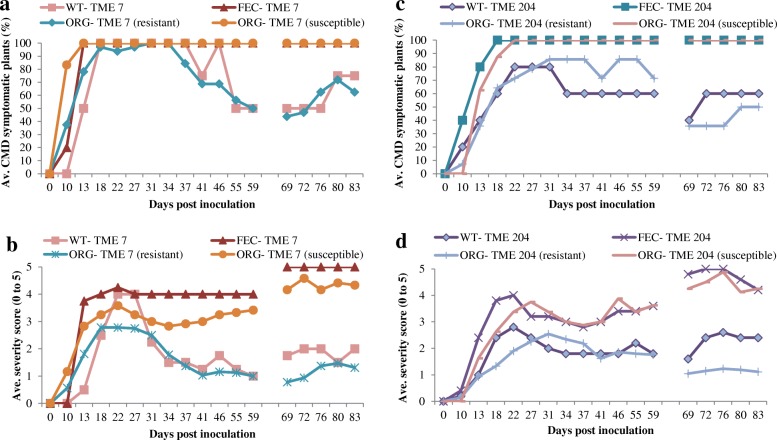
Response of organogenesis-derived plants to inoculation with an infectious geminivirus clone EACMV-K201. **a** Percentage of CMD symptomatic plants of organogenesis-derived (ORG-TME 7) and wild-type CMD2-type cultivar TME 7. **b** Average CMD symptom severity scores (scale 0–5) on organogenesis-derived and wild-type TME 7. **c** Percentage of CMD symptomatic plants of organogenesis-derived (ORG-TME 204) and wild-type CMD2-type cultivar TME 204. **d** Average CMD symptom severity scores (scale 0–5) on organogenesis-derived and wild-type plants of TME 204. Plant stems were cut back at 48 days after biolistic inoculation and CMD was assessed on new leaf growth. Breaks in the *x* axis indicate a lapse in shoot regrowth after cut-back. *n* = 16 for ORG-TME 7 (resistant), *n* = 6 for ORG-TME 7 (susceptible), *n* = 7 for ORG-TME 204 (resistant), *n* = 4 for ORG-TME 204 (susceptible)

We recently described a novel regeneration system in cassava by which plants are recovered from different explant types via caulogenesis. Explants are first cultured on medium containing 1 μM 2,4-D and 1 μM *m*T for 7 days, followed by subculture onto medium supplemented with 6 μM *m*T [11]. Shoots that regenerate on the second-stage medium originate from a hard, dark green colored callus, with no evidence for the occurrence of somatic embryogenesis. Plants of CMD2-type cultivars TME 204 and TME 7 were regenerated from leaf-petiole explants cultured on *m*T [11], established in the greenhouse and inoculated with MeSPY1-VIGS and EACMV-K201 to determine if they had retained resistance to CMD. Loss of resistance to CMD occurred in both cultivars, but only from a portion of the regenerated plant lines. In TME 7, six out of 22 plant lines regenerated through caulogenesis had lost resistance to CMD (Fig. 5a & b; Table 2). Of 11 independent TME 204 regenerant lines inoculated with MeSPY1-VIGS, seven lines were found to have retained resistance, and four to have become susceptible to CMD (Fig. 5c & d; Table 2). All clonal replicates derived from a given regenerated plant line behaved in the same manner, whether resistant or susceptible. When challenged with the EACMV infectious clone ECAMV-K201, the same plant lines from both cultivars remained resistant or susceptible as assessed by their ability to recover from CMD symptoms (Additional file 1: Figure S1).

**Table 2:** Response of organogenesis-derived plants to MeSPY1-VIGS challenge

Cultivar name	No. of dead independent regenerants/total regenerants challenged with MeSPY1-VIGS	Percentage CMD susceptible plants
TME 7	6/22	27
TME 204	4/11	36

**Fig. 6:**
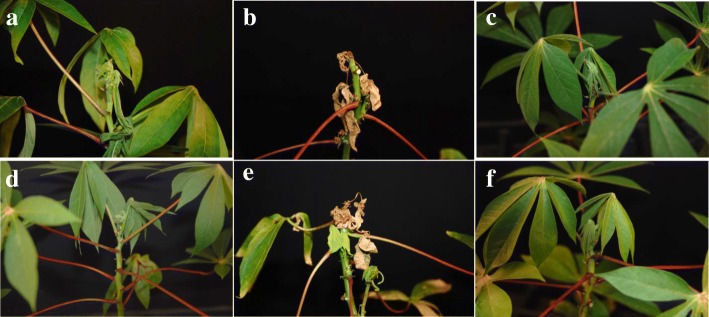
Response of meristem tip culture-derived plants of TME 7, TME 14, TME 7, TMS 98/0505 to inoculation with MeSPY1-VIGS. Silencing of *MeSPY* using MeSPY1-VIGS leads to shoot-tip necrosis and death of the of CMD susceptible cassava plants within 2–4 weeks. **a** CMD resistant micropropagated TME 7 plant. **b** CMD susceptible meristem tip-derived TME 7 plant. **c** CMD resistant meristem tip-derived TME 7 plant. **d** CMD resistant wild-type TME 14 plant. **e** CMD susceptible meristem tip-derived TME 14 plant. **f** CMD resistant meristem tip-derived TME 14 plant

**Fig. 7:**
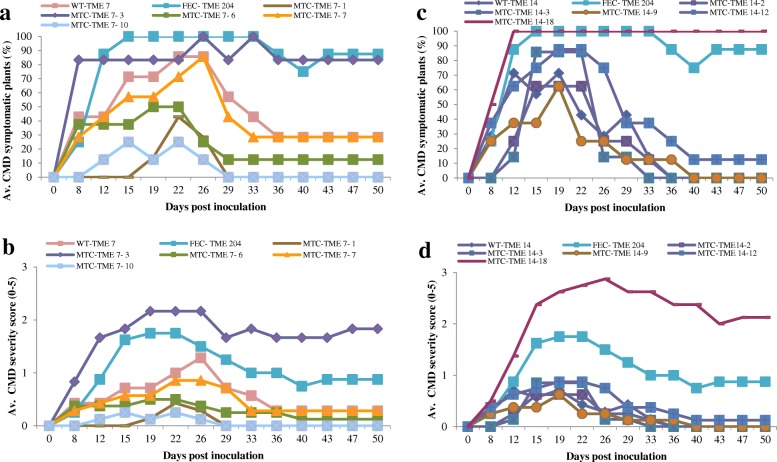
Response of meristem tip-derived plants of cassava to inoculation with infectious geminivirus clone ACMV-CM. **a** Percentage of CMD symptomatic plants of meristem tip-derived CMD2-type cultivar TME 7 plants. **b** Average CMD symptom severity scores (scale 0–5) on meristem tip-derived TME 7 plants. **c** Percentage of CMD symptomatic plants of meristem tip-derived CMD2-type cultivar TME 14 plants. **d** Average CMD symptom severity scores (scale 0–5) on meristem tip-derived TME 14 plants. The FEC-TME 204 (FEC-derived) plants were used as CMD susceptible control and wild-type plants of TME 14 (WT-TME 14) and TME 7 (WT-TME 7) were used as CMD resistant controls. The MTC-TME7 and MTC-TME 14 are meristem tip-derived plants

Meristem tip culture is a well-established method for recovering pathogen-free plants in cassava and many other plant species [22]. The CMD2-type cultivars TME 204, TME 7 and TME 14, the CMD1-type cultivar TMS 30752 and CMD3-type TMS 98/0505 were subjected to meristem tip culture to determine effects of this tissue culture system on CMD resistance (Table 3). Maximum plant regeneration was observed in TME 14 followed by TME 7 and TMS 98/0505. TME 204 showed the lowest shoot regeneration rate with only 12% explants inducing shoots. When inoculated with MeSPY1-VIGS, two out of 17 regenerated plant lines in TME 7 and one out of 19 regenerants in TME 14 were found to have become susceptible to CMD. The remaining plant lines in these and the other cultivars tested retained resistance to CMD, recovering to establish healthy plants (Table 3, Fig. 6). Similar results were obtained when the selected meristem tip-derived plants were challenged with the relatively mild infectious clone ACMV-CM (Fig. 7).

**Table 3:** Response of meristem tip-derived plants to inoculation with MeSPY1 -VIGS challenge

Cultivar name	No. of explants (meristem tip) established^a^	No. of explants forming shoots	Percentage shoot regeneration	No. of dead independent regenerants/total regenerants challenged with MeSPY1-VIGS	Percentage CMD susceptible plants
TME 7	58	28	48	2/17	12
TME 14	40	20	50	1/19	5
TME 204	58	7	12	0/1	0
TMS 30572	18	8	44	0/3	0
TMS 98/0505	58	13	22	0/3	0

^a^Explants were setup in two separate experiments

## Discussion

Morphogenic culture systems are central to the production of transgenic cassava plants and are being adapted for gene editing applications [8, 9]. In many cases, the intention is to deploy the resulting enhanced materials to farmers and/or breeders. Compromised resistance to CMD within such plant lines is therefore a significant concern. Beyene et al. [4] reported that cassava cultivars possessing the dominant, monolocus CMD2-type resistance lost resistance to CMD when passed through somatic embryogenesis. It is essential that full understanding of the developmental and molecular mechanisms underlying loss of resistance to CMD is elucidated. This is required to secure long-term confidence in cassava plants regenerated through tissue culture, to enable improvement of CMD2-type cultivars through genetic engineering and gene-editing technologies, and to understand if and how morphogenic systems could also result in loss of critical traits in other crops. The objectives of the present study were to increase understanding of this phenomenon by screening a wider population of West African elite cassava cultivars for resistance to CMD after somatic embryogenesis, and to determine if alternative morphogenic systems also result in loss of CMD resistance.

Twenty-one cassava cultivars were passed through somatic embryogenesis and subjected to CMD challenge under greenhouse conditions. While CMD2-type cassava became susceptible in the manner reported by Beyene, et al. [4], 15 elite cassava cultivars were confirmed to retain resistance to CMD when regenerated from somatic embryos. FEC produced from TMS 98/0505, TMS 01/0040, TMS 01/0126 and TMS 91/02324 was found to be amenable to *Agrobacterium*-mediated transformation. In all cases, use of moxolactam significantly enhanced production of transgenic tissues and plants. Robust resistance, equivalent to that of the non-modified wild-type plants, was demonstrated in cultivars TMS 98/0505 and TMS 91/02324 after regeneration from all stages of somatic embryogenesis and in transgenic plants of TMS 98/0505. High confidence can be placed, therefore, on the use of existing somatic embryogenesis protocols to introduce desirable traits through transgenic or gene editing technologies in these cultivars.

CMD2-type cultivars are widely grown by farmers in East, West and Central Africa and employed in breeding programs [2, 6, 23]. There is desire to apply biotechnology to improve these varieties for traits including resistance to CBSD [24–26], nutritional enhancement [27, 28] and post-harvest qualities [29] but, as stated above, this must occur without losing the critical trait for CMD resistance. Efforts to develop plant regeneration systems that circumvent the need for somatic embryogenesis resulted in the caulogenic system reported recently by Chauhan and Taylor [11]. In the present study, plants of the CMD2-type cultivars TME 7 and TME 204 regenerated though this cytokinin-based shoot regeneration process were challenged with geminiviruses. In both cultivars, a proportion of the regenerated plant lines were confirmed to have lost resistance to CMD. This response was uniform and stable across clonal replicates of a given line, such that all plants of a regenerated resistant line remained resistant, and those that were susceptible remained susceptible.

Data from plants regenerated through caulogenesis and meristem tip culture provide clear evidence that somatic embryogenesis *per se* is not the underlying cause for loss of resistance to geminiviruses. Unlike somatic embryogenesis, shoot regeneration using meta-topolin does not involve exposure of tissues to high levels of auxin, nor the somaclonal variation associated with such culture systems. It remains unknown how loss of resistance occurs, and why 27–36% of plant lines regenerated via caulogenesis lost resistance, while others remained fully resistant. A possible explanation is that disruption of shoot meristem integrity may be an underlying contributor to loss of CMD resistance in CMD2-type regenerated plants. To test this hypothesis, meristem tip culture was investigated in CMD2-type cultivars. In this case, a small percentage (5–12%) of plant lines regenerated from both CMD2-type cultivars (TME 7 and TME 14) were found to have lost resistance to CMD. These plants were susceptible even to a relatively less virulent strain of ACMV-CM, in the same manner described previously for somatic embryo-derived plants [4]. An alternative hypothesis is that epigenetic changes occur as a result of morphogenesis in CMD2-type cassava cultivars. Such changes may affect resistance gene(s) and/or susceptibility genes at the CMD2 locus. Indeed, it has previously been shown in five cassava cultivars that the meristem-derived plants were epigenetically different than the field grown plants [5]. Additional studies are underway to test these hypotheses.

## Conclusions

The information presented here has important implications for biotechnological applications in cassava, and efforts to elucidate mechanisms of resistance to CMD. Non-CMD2-type cultivars are not affected by passage through somatic embryogenesis or other morphogenic systems, and can therefore be used with confidence as targets for transgenic and gene editing enhancement and mass propagation through tissue culture. Secondly, regeneration via caulogenesis provides a potential solution for generating modified CMD2-type cultivars that retain resistance to CMD. However, plants regenerated in this manner require testing for their resistance to geminiviruses to eliminate those that have been compromised. Finally, meristem tip culture should be used with caution when applied to cassava cultivars carrying the CMD2-type mechanism because it is possible to lose resistance to CMD in plants recovered through this regeneration system. As for caulogenesis, regenerated plant lines should be tested empirically to confirm that CMD resistance is fully functional before dissemination to farmers or establishment in germplasm collections. Loss of resistance through three culture systems provides a powerful toolset for investigating the molecular mechanism behind CMD resistance. The information described here will be critical for designing experiments and interpreting genomic, transcriptomic and epigenomic datasets focused on such efforts.

## Additional file

Additional file 1: **Figure S1.** Response of organogenesis-derived plants of cassava to inoculation with an infectious geminivirus clone of EACMV-K201 and MeSPY1–VIGS. **a** EACMV**-**K201 (left) and MeSPY1-VIGS (right) challenged plants of micropropagated TME 7. **b** EACMV**-**K201 (left) and MeSPY1-VIGS (right) challenged FEC-derived plants of TME 7. **c & d** EACMV**-**K201 (left) and MeSPY1-VIGS (right) challenged organogenesis-derived plants of TME 7. **e** EACMV**-**K201 (left) and MeSPY1-VIGS (right) challenged plants of micropropagated TME 204. **f** EACMV**-**K201 (left) and MeSPY1-VIGS (right) challenged FEC-derived plants of TME 204. **g & h **EACMV**-**K201 (left) and MeSPY1-VIGS (right) challenged organogenesis-derived plants of TME 204. (PPTX 72459 kb)
