# Supplementary material for: Multiple morphogenic culture systems cause loss of resistance to cassava mosaic disease
Source: BMC Plant Biol. 2018 Jun 25;18:132. doi: 10.1186/s12870-018-1354-x (PMC6020238; doi:10.1186/s12870-018-1354-x)
Supplement: Supplementary file 1 — Figure S1. Response of organogenesis-derived plants of cassava to inoculation with an infectious geminivirus clone of EACMV-K201 and MeSPY1–VIGS. a EACMV-K201 (left) and MeSPY1-VIGS (right) challenged plants of micropropagated TME 7. b EACMV-K201 (left) and MeSPY1-VIGS (right) challenged FEC-derived plants of TME 7. c & d EACMV-K201 (left) and MeSPY1-VIGS (right) challenged organogenesis-derived plants of TME 7. e EACMV-K201 (left) and MeSPY1-VIGS (right) challenged plants of micropropagated TME 204. f EACMV-K201 (left) and MeSPY1-VIGS (right) challenged FEC-derived plants of TME 204. g & h EACMV-K201 (left) and MeSPY1-VIGS (right) challenged organogenesis-derived plants of TME 204. (PPTX 72459 kb) [file 12870_2018_1354_MOESM1_ESM.pptx]

## Slide 1
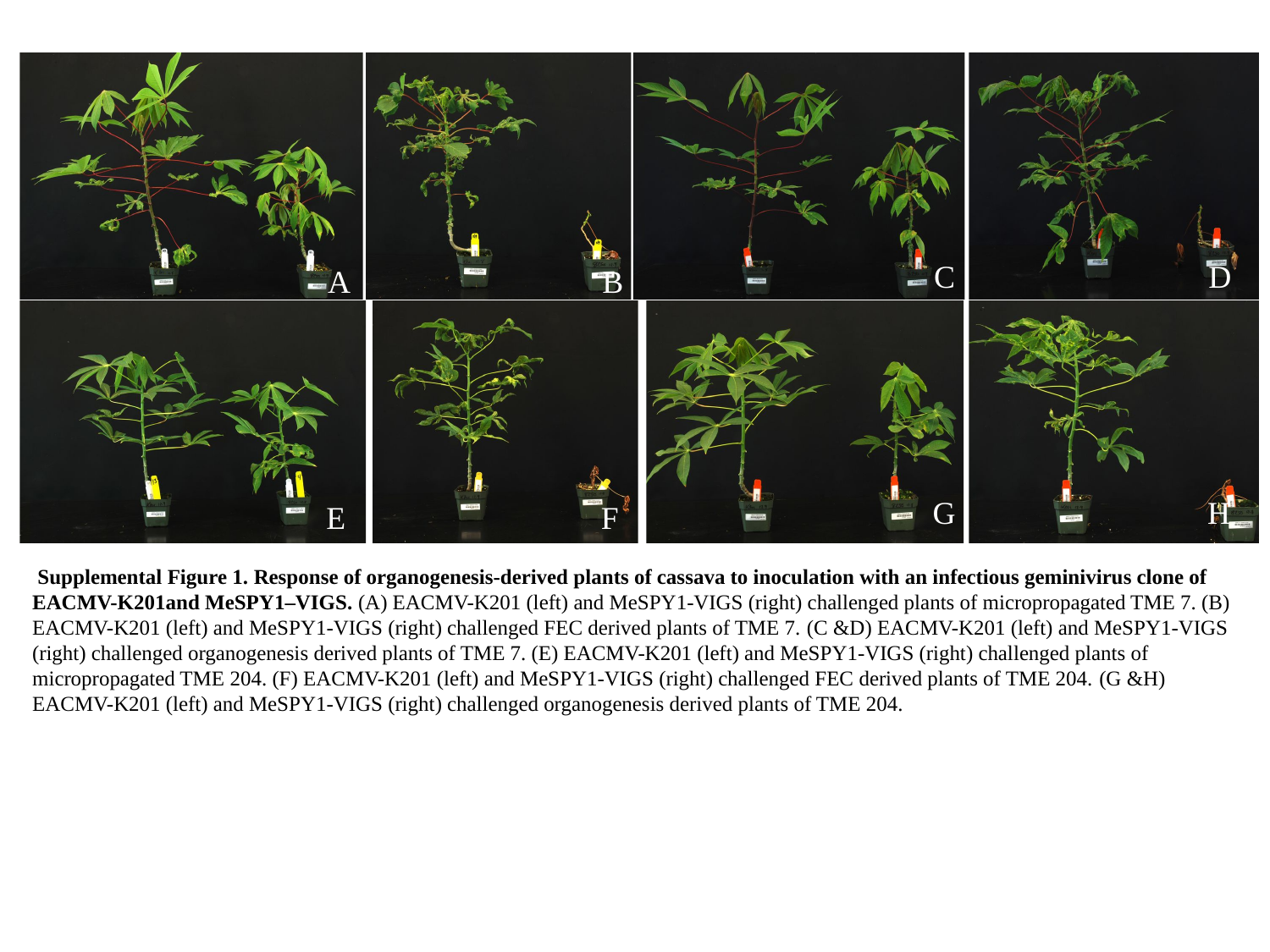

C
D
A
B
G
H
E
F
 Supplemental Figure 1. Response of organogenesis-derived plants of cassava to inoculation with an infectious geminivirus clone of EACMV-K201and MeSPY1–VIGS. (A) EACMV-K201 (left) and MeSPY1-VIGS (right) challenged plants of micropropagated TME 7. (B) EACMV-K201 (left) and MeSPY1-VIGS (right) challenged FEC derived plants of TME 7. (C &D) EACMV-K201 (left) and MeSPY1-VIGS (right) challenged organogenesis derived plants of TME 7. (E) EACMV-K201 (left) and MeSPY1-VIGS (right) challenged plants of micropropagated TME 204. (F) EACMV-K201 (left) and MeSPY1-VIGS (right) challenged FEC derived plants of TME 204. (G &H) EACMV-K201 (left) and MeSPY1-VIGS (right) challenged organogenesis derived plants of TME 204.
